# Association Between Nutritional Status and Extranodal Extension of Lymph Node Metastases in Head and Neck Squamous Cell Cancers

**DOI:** 10.3390/nu18040706

**Published:** 2026-02-22

**Authors:** Kornél Dános, Mátyás Majoros, Lili Tóth, Benedek Besenczi, Mohammad Aouf, Angéla Horváth, László Tamás, Imre Uri

**Affiliations:** 1 Department of Oto-Rhino-Laryngology, Head and Neck Surgery, Semmelweis University, 1085 Budapest, Hungary; danos.kornel@semmelweis.hu (K.D.);; 2 Otorhinolaryngology Department, Faculty of Medicine, Kafr El-Sheikh University, Kafr El-Sheikh 33516, Egypt; 3 Department of Voice, Speech, and Swallowing Therapy, Semmelweis University, 1085 Budapest, Hungary

**Keywords:** prognostic nutritional index (PNI), body mass index (BMI), extranodal extension (ENE), head and neck squamous cell carcinoma (HNSCC)

## Abstract

Introduction: Extranodal extension (ENE) is a well-established adverse prognostic factor in head and neck squamous cell carcinoma (HNSCC), associated with reduced survival and the need for intensified therapy. Nutritional status—commonly assessed using the Prognostic Nutritional Index (PNI) and Body Mass Index (BMI)—also influences outcomes in HNSCC. However, whether or not ENE correlates with nutritional status has not been previously investigated. Methods: We conducted a retrospective cohort study of 109 treatment-naïve HNSCC patients with pathologically confirmed nodal metastases who underwent primary tumor resection and neck dissection between 2014 and 2025 at a national tertiary center. ENE status was determined histologically. Nutritional status was evaluated using BMI, PNI, serum albumin, and percentage of weight loss at diagnosis. Statistical analyses included *t*-tests, Chi-square tests, ANOVA, Cox regression, Kaplan–Meier survival analysis, and Full Factorial General Linear Models. Results: ENE was present in 54.1% of patients and significantly reduced overall survival (Kaplan–Meier *p* = 0.006; Cox regression RR = 1.927, *p* = 0.008). No significant differences in BMI, PNI, weight loss, or serum albumin were observed between ENE-positive and ENE-negative groups. ENE prevalence varied significantly by tumor origin (*p* = 0.018), being highest in hypopharyngeal cancers (75.8%) and lowest in oral cavity tumors (25.0%). ENE status was independent of tobacco use, alcohol abuse, and all nutritional markers across TNM 8/9 subgroups. Conclusions: ENE is a strong prognostic marker in HNSCC, appearing to be independent of nutritional status. The demonstrated heterogeneity of ENE prevalence among tumor subsites supports the need for individualized management approaches.

## 1. Introduction

Extranodal extension (ENE) of head and neck squamous cell cancer (HNSCC) represents one of the most significant prognostic markers in oncology. It is independently associated with decreased overall survival (OS), disease-specific survival (DSS), and disease-free survival (DFS) [1,2,3]. Reflecting its profound negative prognostic impact, the Union for International Cancer Control (UICC) 8th TNM Edition introduced ENE assessment as a mandatory component of patient workup. ENE-positivity necessitates more aggressive treatment decisions, including adjuvant radiotherapy with concurrent chemotherapy as supported by landmark trials such as RTOG 9501 and EORTC 22931 [4,5]. The clinical significance of ENE has only grown stronger over time; the 9th TNM Edition now reintroduces ENE into the staging of p16-positive oropharyngeal cancer (OPC), further underscoring its importance in risk stratification [6,7,8].

While ENE is a well-established adverse prognostic factor, the biological mechanisms underlying its development and potential modifiable risk factors remain incompletely understood. Recent literature highlights significant heterogeneity in ENE rates across tumor subsites—hypopharyngeal cancers exhibit dramatically higher ENE rates than oral cavity cancers—suggesting that ENE development may be driven by distinct biological processes that depend on tumor origin [9,10,11,12,13,14,15].

Nutritional status is an increasingly recognized independent prognostic factor in HNSCC. Better nutritional status has been shown to provide a significant survival benefit among HNSCC patients, influencing not only treatment tolerability and completion rates but also long-term oncologic outcomes. The literature identifies the Prognostic Nutritional Index (PNI)—a composite measure integrating serum albumin concentration and lymphocyte count—as the most reliable screening tool for malnutrition in HNSCC. Unlike Body Mass Index (BMI), which fails to account for sarcopenia across all BMI categories, the PNI reflects a dual immune-nutritional mechanism that captures both nutritional adequacy and immune competence. Serum albumin, as a negative acute-phase protein and marker of amino acid sufficiency, reflects systemic inflammatory status and protein synthesis capacity. Pre-treatment weight loss, though generally weaker in predictive strength than composite indices, remains an established adverse prognostic indicator [9,10,11,12,13,14,15].

Extensive evidence demonstrates that advanced tumor stage correlates with deteriorated nutritional status. Tumors in advanced stages (III/IV) cause dysphagia, odynophagia, and trismus through mass effect or direct invasion, reducing oral intake and precipitating pre-treatment malnutrition. Up to 70% of advanced HNSCC patients present clinical malnutrition at diagnosis. Additionally, systemic inflammation and cancer cachexia accelerate muscle wasting through elevated cytokines, independent of reduced caloric intake. Similarly, nodal positivity has been associated with poorer nutritional status due to increased tumor burden affecting swallowing function, lymphatic obstruction, and pain-related dysphagia. One emerging line of evidence suggests that nodal metastasis may exacerbate nutritional decline by modulating immune function, as node-positive patients demonstrated significantly elevated neutrophil-to-lymphocyte ratios [9,10,11,12,13,14,15].

ENE represents an aggressive biological phenotype with worse survival, nutritional status reflects immune competence and metabolic health, and nodal involvement (related to ENE development) correlates with nutritional decline. Despite the well-established prognostic importance of both ENE and nutritional status as independent factors in HNSCC, the relationship between these two critical variables has never been investigated.

To address this critical gap in the literature, we conducted a study of treatment-naïve HNSCC patients with pathologically confirmed nodal metastases to investigate whether ENE status correlates with nutritional status, as assessed by multiple markers: Body Mass Index, Prognostic Nutritional Index, serum albumin, and percentage weight loss at diagnosis.

## 2. Patients and Methods

We conducted a retrospective cohort study of patients diagnosed and treated for head and neck squamous cell carcinoma (HNSCC) between 2014 and 2025 at the Department of Oto-Rhino-Laryngology, Head and Neck Surgery, Semmelweis University, Budapest, Hungary. Inclusion criteria were as follows: patients who underwent primary surgical treatment, including neck dissection, and whose tumors originated in the oral cavity, oropharynx, hypopharynx, or the supraglottic, glottic, or subglottic regions of the larynx. Pathological examination had to confirm metastatic involvement of cervical lymph nodes. All patients meeting these criteria within the study period were included.

In cases of oropharyngeal cancers, p16-status was recorded, all tumors were (re)classified according to the UICC 8th TNM classification.

As a tertiary referral center treating patients from across the country, our cohort has the potential to represent the Hungarian HNSCC population.

Only patients with proven neck metastasis who underwent primary neck dissection without prior treatment (irradiation or systemic therapy) were included. Pathological ENE status was determined by histological examination.

In all cases, pathological ENE was assessed based on the criterion of tumor extension beyond the nodal capsule. Surgical specimens were processed and examined by the Department of Pathology, Forensic and Insurance Medicine at Semmelweis University.

Nutritional status was evaluated using BMI, PNI, serum albumin level, and percent of weight loss over the last six months. PNIs were calculated as: Serum albumin (g/L) + 5 × Lymphocyte count (G/L). Laboratory measurements were performed as part of the diagnostic workup around the time of tumor biopsy, which we defined as the time of diagnosis.

Survival data were confirmed using the Hungarian National Cancer Registry.

Statistical analyses included Log-Rank Tests, Cox regression, Kaplan–Meier curves, and logistic regression. As most of the nutritional variables did follow normal distribution and the sampling was with high element number (Shapiro–Wilk test, histogram inspection, analysis of skewness and kurtosis, Central Limit Theorem), we used independent sample t-tests after conducting Levene’s test for equality of variances. For crosstabulations, Pearson’s Chi-square and Yates corrected Chi-square tests were applied. Group comparison was performed using one-way ANOVA and Tukey HSD post hoc test. Full Factorial General Linear Model was used to eliminate potential confounding effect of tumor origin. Missing data were handled using pairwise deletion to maximize the available sample size for each statistical comparison. The significance level was set at 5%. Analyses were performed using TIBCO Statistica 14.0.

## 3. Results

A total of 109 patients met the inclusion criteria. Descriptive statistics are presented in Table 1 and Table 2.

**Figure 1:**
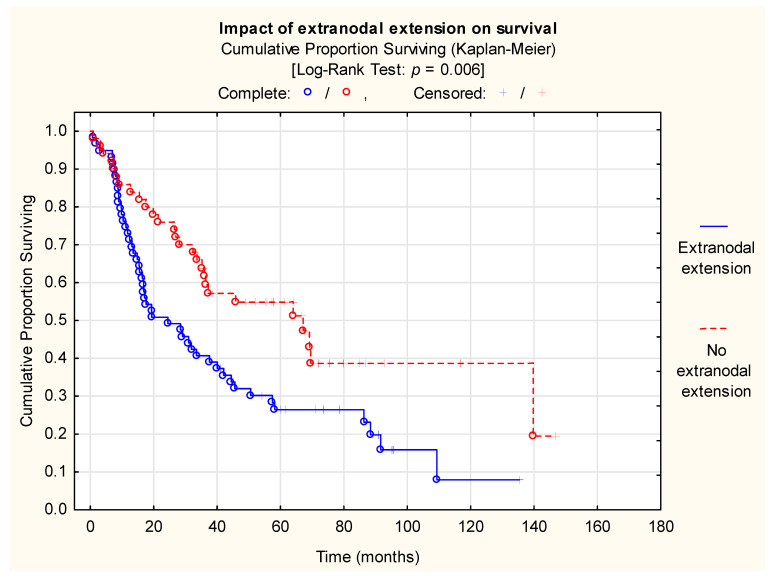
Impact of extranodal extension on survival (Log-Rank Test: WW = 11.554, Sum = 71.913, Var = 18.021, Test statistic = 2.722, *p* = 0.006, Power (α = 0.05) = 0.780).

ENE had a significant impact on overall survival (*p* = 0.006, Kaplan–Meier curve; Figure 1). Cox regression confirmed this finding: ENE-positivity nearly doubled the risk of death (*p* = 0.008, RR = 1.927, Table 3).

**Figure 2:**
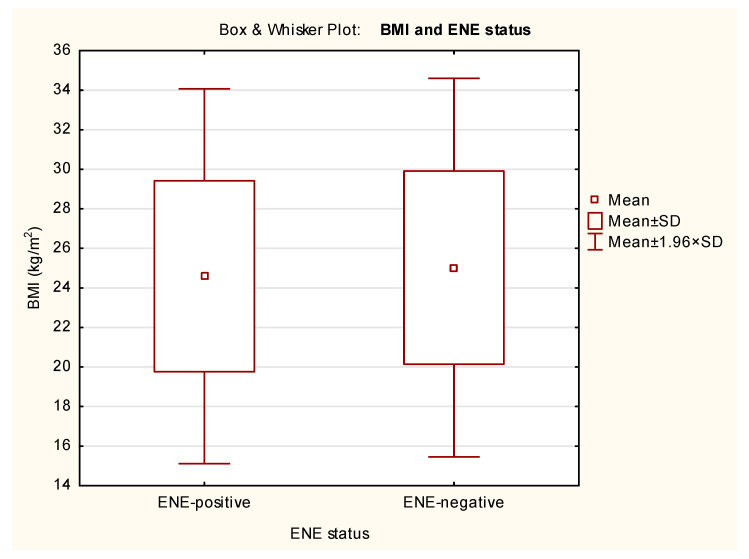
Box and Whisker Plot of BMI in relation to ENE status. The means, means ± standard deviations (SD), and the 95% confidence intervals (mean ± 1.96 × SD) are shown.

**Figure 3:**
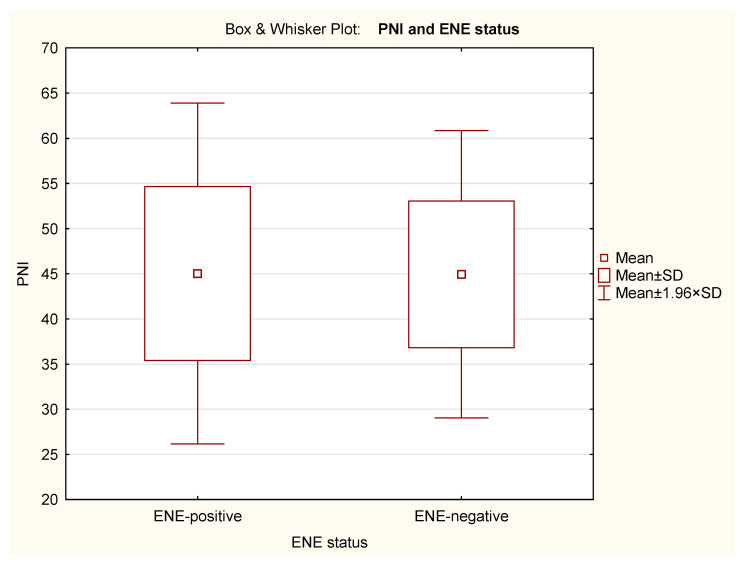
Box and Whisker Plot of PNI in relation to ENE status. The means, means ± standard deviations (SD), and the 95% confidence intervals (mean ± 1.96 × SD) are shown.

**Figure 4:**
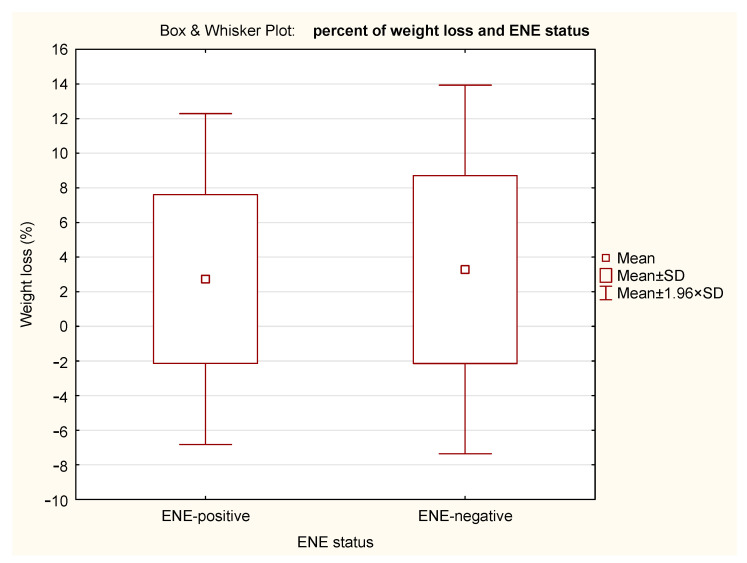
Box and Whisker Plot of weight loss in relation to ENE status. The means, means ± standard deviations (SD), and the 95% confidence intervals (mean ± 1.96 × SD) are shown.

**Figure 5:**
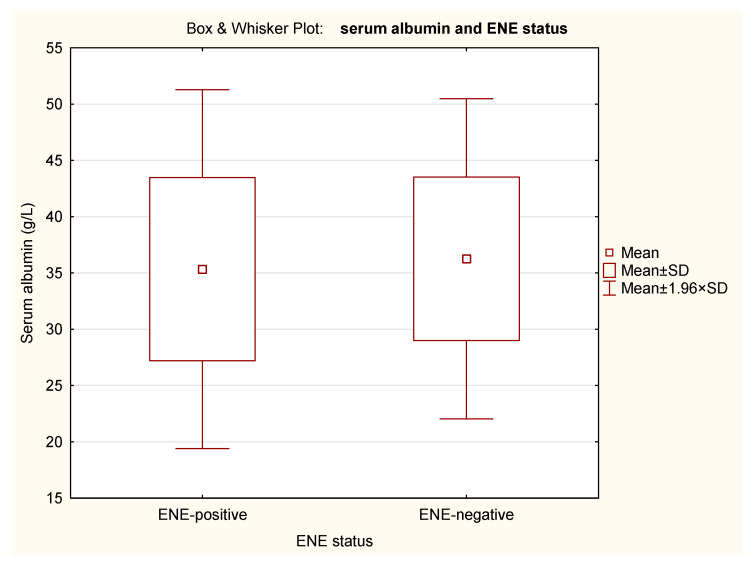
Box and Whisker Plot of serum albumin in relation to ENE status. The means, means ± standard deviations (SD), and the 95% confidence intervals (mean ± 1.96 × SD) are shown.

ENE did not significantly affect BMI (*p* = 0.649), PNI (*p* = 0.975), or percentage of weight loss (*p* = 0.606) of albumin (*p* = 0.606) in our cohort (Table 4). The Box and Whisker Plots with BMI, PNI, percent of weight loss and serum albumin in relation to ENE status are presented in Figure 2, Figure 3, Figure 4 and Figure 5.

Pearson’s Chi-square test showed significant differences in ENE distribution among TNM 8/9 subgroups (*p* = 0.018). ENE was most common in hypopharyngeal cancer (75.8%) and least common in oral cavity cancer (25.0%) (Table 5).

One-way ANOVA showed no significant differences in PNI (*p* = 0.317), weight loss (*p* = 0.115) or serum albumin (*p* = 0.435) among TNM 8/9 groups, but BMI differed significantly (*p* = 0.047). Post hoc Tukey HSD testing identified significant difference only between p16-positive and p16-negative oropharyngeal cancers (*p* = 0.030) (Table 6).

To bypass the possible confounding effect of TNM 8/9 groups, we performed a Full Factorial General Linear Model, with tumor origin as categorical factor. The analysis confirmed that ENE status was independent of BMI (*p* = 0.527), PNI (*p* = 0.572), weight loss (*p* = 0.225), and albumin (*p* = 0.365) across all TNM groups (Table 7).

Yates’s corrected Chi-square tests showed no significant association between ENE status and tobacco use (*p* = 0.731) or alcohol abuse (*p* = 0.239) (Table 8).

## 4. Discussion

The importance of extranodal extension in HNSCC—including cancers of the oral cavity, pharynx, and larynx—is increasingly recognized. The UICC TNM classification expanded ENE evaluation: while it was absent from the 7th Edition, the 8th Edition introduced ENE as a prognostic factor, upstaging clinical and pathological nodal disease to N3b, except in p16-positive-oropharyngeal cancers [16]. The 9th Edition extends ENE staging to this subgroup and refines imaging-detected ENE criteria for clinical and pathological nodal staging [17,18].

The urge to emphasize ENE staging was supported by a study of 2053 patients, which found that multivariable analysis identified ENE as the strongest prognostic nodal feature in p16-positive OPC (adjusted HR for imaging-detected ENE: 2.43) [19].

Given its poor prognostic implications, ENE-positivity necessitates more aggressive oncologic management. In ENE-positive cases, adjuvant radiotherapy with concurrent chemotherapy is supported by landmark trials, such as RTOG 9501 and EORTC 22931 [4,5]. Surgically, ENE may necessitate more extensive neck dissection, influence resection margins and reconstruction planning, or render the disease unresectable, shifting patients toward palliative treatment.

Pathological and radiological ENE evaluation required clarification due to methodological differences. The International Consensus Recommendations define ENE as “extension of viable tumor outside the lymph node capsule into the perinodal soft tissues with or without a desmoplastic reaction” [2]. ENE creates anatomical challenges for disease clearance, increasing the risk of recurrence and distant metastasis [20].

Literature distinguishes minor/microscopic ENE (≤2 mm beyond the capsule) from major/macroscopic ENE (>2 mm). The prognostic significance of this distinction remains controversial: some studies report differences in survival [21,22], while others do not [23,24]. Some authors recommend a 5 mm cutoff for oral cavity cancers [25]. Further research is required to clarify its impact. TNM 8 and 9 do not differentiate between minor and major ENE. Due to the retrospective nature of our study, we do not have access to sufficient data regarding ENE extent.

The ENE examination is a rapidly evolving area in head and neck risk stratification, an area that still holds many secrets to be discovered. The correlation between nutritional status and extranodal extension has not yet been investigated.

Tumor stage is a known predictor of nutritional risk, as advanced T stages increase vulnerability through catabolism, reduced appetite, swallowing dysfunction, xerostomia, and severe odynophagia/dysphagia [26,27]. Nodal positivity also correlates with poorer nutritional status due to increased tumor burden affecting swallowing, lymphatic obstruction, pain, and dysphagia [28]. One study suggested that nodal metastasis may exacerbate nutritional decline by modulating the immune system, as node-positive patients had significantly elevated neutrophil-to-lymphocyte ratios (NLR) [29]. We aimed to investigate whether ENE-positivity influences nutritional status.

Nutritional assessment is essential in patient workup, as it affects ECOG performance status, fitness for curative (chemo)radiotherapy or surgery, and wound healing [30]. Adequate nutrition also improves the OS of patients with recurrent/metastatic HNSCC [31]. In our cohort, ENE-positive patients had significantly shorter OS (log-rank *p* = 0.006; Cox *p* = 0.008, RR = 1.927), consistent with the literature.

The reviewed literature emphasizes the significance of BMI and PNI as tools for evaluating nutritional status. BMI, derived from anthropometric measurements (height and body weight), remains a widely utilized indicator. Nevertheless, BMI has limited utility, as it fails to account for sarcopenia, which may manifest across all BMI categories [32]. Conversely, PNI reflects a dual immune-nutritional mechanism. It is calculated using serum albumin concentration and lymphocyte count, thereby integrating markers of both immune competence and nutritional status. Malnutrition impairs lymphopoiesis, while chronic inflammation accelerates lymphocyte apoptosis [33].

Serum albumin can be assessed either as an individual parameter or as a component of the PNI calculation. It serves as a multifaceted indicator of malnutrition: its synthesis declines in the context of amino acid deficiency, while hypermetabolic states enhance its catabolism. Reduced levels signify systemic inflammation, given its role as a negative acute-phase protein [15,34]. Current evidence identifies PNI as the most reliable screening tool for malnutrition in patients with HNSCC [9,10,11,12,13].

Pre-treatment weight loss is a recognized adverse prognostic factor in HNSCC, although its predictive strength is generally weaker than that of composite nutritional–inflammatory indices such as the PNI [13]; nevertheless, the findings related to weight loss further supported our conclusion that nutritional status and ENE positivity are independent of one another.

As nutritional status affects several protecting mechanisms (e.g., the immune system), we hypothesized that, due to this, ENE-positivity is more common among patients with poor nutritional status. In view of these considerations, both BMI and PNI were used to assess nutritional status. t-tests showed no significant differences between ENE-positive and ENE-negative patients (BMI *p* = 0.649; PNI *p* = 0.975; weight loss *p* = 0.606; serum albumin *p* = 0.606).

Different tumor origins (TNM8/9 subgroups) have characteristic nodal metastatic potential [35,36,37]. However, no studies have examined their potential for developing ENE. Our Pearson Chi-square analysis demonstrated significant differences in ENE distribution among TNM8/9 subgroups (*p* = 0.018). Hypopharyngeal cancers had the highest ENE rate (75.8%), followed by supraglottic laryngeal (58.3%), glottic laryngeal (50.0%), p16-negative oropharyngeal (44.4%), p16-positive oropharyngeal (30.0%), and oral cavity cancers (25.0%). Because tumor origin could act as a confounding factor when assessing the relationship between ENE and nutritional status, we conducted additional analyses to control its potential influence.

One-way ANOVA showed significant differences in BMI among TNM8/9 groups (*p* = 0.047), but not in PNI (*p* = 0.317), weight loss (*p* = 0.115) or serum albumin (*p* = 0.435). Full Factorial General Linear Model analysis confirmed that ENE status was independent of BMI, PNI, weight loss and serum albumin across all TNM8/9 subgroups.

We found no relevant literature on ENE and tobacco/alcohol use. Yates corrected Chi-square tests showed no association between ENE status and tobacco (*p* = 0.731) or alcohol abuse (*p* = 0.239).

## 5. Conclusions

Extranodal extension (ENE) is one of the most powerful prognostic factors in HNSCC, exerting a survival impact that is independent of tumor stage and increasingly central to diagnostic and therapeutic decision-making. Nutritional status has likewise emerged as a prognostic determinant, yet its relationship with ENE has not previously been examined. We explored this connection as a novel research question. Our findings demonstrate that, unlike tumor stage or nodal size, ENE is independent of nutritional status as assessed by BMI, PNI, six-month weight loss, and serum albumin. Given that nutritional status itself carries prognostic significance, it should remain an integral component of routine patient evaluation and treatment planning, complementing TNM-based risk stratification rather than interacting with ENE.

Major etiologic factors such as tobacco and alcohol abuse were also unrelated to ENE status in our cohort. In contrast, ENE prevalence varied significantly across tumor subsites defined by the TNM 8/9 classifications, underscoring biological heterogeneity in metastatic behavior.

The primary limitations of this study include its retrospective design and moderate cohort size, both of which limit the statistical power to detect subtle associations. Nevertheless, the real-world nature of the dataset—comprising all eligible patients treated at a national tertiary center—supports the representativeness of our findings for the Hungarian HNSCC population. Prospective, larger-scale studies are warranted to validate these observations and further clarify the biological determinants of ENE.

## Figures and Tables

**Table 1:** Descriptive statistics. The grouping criteria are highlighted in bold.

	Total	ENE-Negative	ENE-Positive
**all groups**	109	50	59
			
**TNM 8/9 Group**			
oral cavity	12	9	3
p16 neg. oropharynx	18	10	8
p16 pos. oropharynx	10	7	3
hypopharynx	33	8	25
supraglottic larynx	12	6	6
glottic larynx	24	10	14
Missing	0	0	0

**T stage**			
1	11	8	3
2	36	13	23
3	23	15	8
4	39	14	25
Missing	0	0	0

**ECOG**			
0	65	35	30
1	30	13	17
2	5	1	4
Missing	9	1	8

**tobacco**			
no	9	4	5
yes	97	43	54
Missing	3	3	0

**alcohol**			
no	42	23	19
yes	61	25	36
Missing	6	2	4

**Table 2:** Descriptive statistics (BMI = Body Mass Index, PNI = Prognostic Nutritional Index).

		Total	ENE-Positive	ENE-Negative
BMI (kg/m^2^)	Valid N	103	55	53
	Mean	24.795	24.591	25.259
	Std.Dev.	24.221	24.221	4.819
	Median	15.699	15.699	24.307
	Maximum	37.751	37.751	17.637
	Minimum	4.840	4.836	35.640
	Skewness	0.534	0.474	0.522
	Kurtosis	−0.299	−0.020	−0.670
	K-S test (*p*)	>0.200	>0.200	>0.200
PNI	Valid N	46	31	15
	Mean	45.002	45.032	44.940
	Std. Dev.	43.725	43.800	8.125
	Median	28.450	28.450	43.650
	Maximum	62.150	62.150	31.000
	Minimum	9.078	9.634	55.350
	Skewness	−0.179	−0.139	−0.363
	Kurtosis	−0.938	−0.957	−1.073
	K-S test (*p*)	>0.200	>0.200	>0.200
Weight loss (%)	Valid N	93	51	42
	Mean	2.982	2.732	3.285
	Std. Dev.	5.112	4.875	5.431
	Median	0.000	0.000	0.000
	Maximum	24.390	24.390	19.355
	Minimum	0.000	0.000	0.000
	Skewness	2.049	2.326	1.836
	Kurtosis	4.253	6.868	2.577
	K-S test (*p*)	<0.010	<0.010	<0.010
Serum albumin (g/L)	Valid N	46	31	15
	Mean	35.641	35.342	36.260
	Std. Dev.	36.000	35.700	39.800
	Median	21.500	21.500	27.300
	Maximum	50.200	50.200	44.800
	Minimum	7.791	8.135	7.258
	Skewness	−0.035	0.007	−0.089
	Kurtosis	−1.207	−1.025	−2.117
	K-S test (*p*)	>0.200	>0.200	>0.200
Age (years) at the time of diagnosis	Valid N	109	59	50
	Mean	61.929	62.834	60.861
	Std. Dev.	61.982	6.888	9.271
	Median	42.675	62.943	60.569
	Maximum	76.753	76.753	76.016
	Minimum	8.090	43.376	42.675
	Skewness	−0.305	−0.265	−0.166
	Kurtosis	−0.350	0.183	−0.845
	K-S test (*p*)	>0.200	>0.200	>0.200
Overall survival in months	Valid N	109	59	50
	Mean	42.170	36.940	48.341
	Std. Dev.	32.931	32.195	33.039
	Median	35.500	24.467	42.233
	Maximum	146.800	135.367	146.800
	Minimum	32.931	0.733	1.000
	Skewness	0.962	1.068	0.940
	Kurtosis	0.607	0.358	1.183
	K-S test (*p*)	<0.100	<0.050	>0.200

**Table 3:** Cox regression of ENE-positive/negative status. (RR = Risk ratio, CI = confidence interval).

Dependent Variable: Overall Survival (Months), Censoring Var.: Died (1/0), Chi2 = 7.473, df = 1, *p* = 0.006, Power (α = 0.05) = 0.880										
N = 109	Beta	Stand. Error	Beta CI −95%	Beta CI +95%	t-Value	Wald Statist.	*p*	Risk Ratio	RR CI −95%	RR CI +95%
ENE +/−	0.656	0.245	0.175	1.137	2.672	7.140	0.008	1.927	1.191	3.118

**Table 4:** Independent sample t-test of nutritional status markers. (CI = confidence interval, ΔM = Mean [ENE+] − Mean [ENE−]). The test headers are highlighted in bold.

Independent Sample *t*-Test, Grouping: ENE +/− (Equal Variance Estimated)										
Variable	Valid N (ENE+)	Valid N (ENE−)	Mean (ENE+)	Mean (ENE−)	t	df	*p*	M(ENE+) − M(ENE−)	ΔM CI −/+95%	Power (α = 0.05)
BMI	55	48	24.590	25.028	−0.456	101	0.649	−0.438	−2.342–1.466	0.120
PNI	31	15	45.032	44.940	0.032	44	0.975	0.092	−5.727–5.912	0.060
weight loss	51	42	2.732	3.285	−0.517	91	0.606	−0.553	−2.677–1.571	0.140
albumin	31	15	35,342	36,260	−0.371	44	0.712	−0.918	−9.974–4.069	0.100
										
**F-test and Levene-test**										
**Variable**	**Std. Dev. (ENE+)**	**Std. Dev. (ENE-)**	**F-ratio (Variances)**	***p*** **(Variances)**	**Levene (F(1.df))**	**df (Levene)**	***p*** **(Levene)**			
BMI	4.836	4.885	1.021	0.937	0.054	101.000	0.817			
PNI	9.634	8.125	1.406	0.507	0.282	44.000	0.598			
weight loss	8.135	7.258	1.256	0.667	0.019	44	0.891			
albumin	7.899	6.797	1.351	0.461	0.161	58	0.690			

**Table 5:** Crosstabulation and Pearson Chi-square test of ENE status and TNM 8/9 groups.

2-Way Summary Table: Observed Frequencies, Pearson Chi-Square Test. Power (α = 0.05) = 0.720			
	ENE-Negative	ENE-Positive	Row TOTALS
oral cavity	9 (75.0%)	3 (25.0%)	12
p16 neg. oropharynx	10 (55.6%)	8 (44.4%)	18
p16 pos. oropharynx	7 (70.0%)	3 (30.0%)	10
hypopharynx	8 (24.2%)	25 (75.8%)	33
supraglottic larynx	6 (50.0%)	6 (50.0%)	12
glottic larynx	10 (41.7%)	14 (58.3%)	24
Totals	50	59	109
Pearson Chi-square	Chi-square = 13.596	df = 5	*p* = 0.018

**Table 6:** One-way ANOVA of BMI, PNI, weight loss, albumin and TNM 8/9 subgroup, Tukey HSD with BMI. The test headers are highlighted in bold.

**One-Way ANOVA: Univariate Results for Each DV. Sigma-Restricted Parameterization. Effective Hypothesis Decomposition. Power (α = 0.05) = 0.780**						
**Effect**	**Degr. of Freedom**	**BMI** **SS**	**BMI** **MS**	**BMI** **F**	**BMI** ***p***	
Intercept	1	53,420.740	53,420.740	2431.220	<0.001	
TNM group	5	258.020	51.600	2.349	0.047	
Error	97	2131.360	21.970			
Total	102	2389.380				
**One-way ANOVA: Univariate Results for Each DV. Sigma-restricted parameterization. Effective hypothesis decomposition. Power (α = 0.05) = 0.580**						
**Effect**	**Degr. of Freedom**	**PNI** **SS**	**PNI** **MS**	**PNI** **F**	**PNI** ***p***	
Intercept	1	43,481.940	43,481.940	540.548	<0.001	
TNM group	5	490.870	98.170	1.220	0.317	
Error	40	3217.620	80.440			
Total	45	3708.490				
**One-way ANOVA: Univariate Results for Each DV. Sigma-restricted parameterization. Effective hypothesis decomposition. Power = 0.700**						
**Effect**	**Degr. of Freedom**	**Weight loss** **SS**	**Weight loss** **MS**	**Weight loss** **F**	**Weight loss** ***p***	
Intercept	1	671.770	671.770	26.864	<0.001	
TNM group	5	228.945	45.789	1.831	0.115	
Error	87	2175.519	25.006			
**One-way ANOVA: Univariate Results for Each DV. Sigma-restricted parameterization. Effective hypothesis decomposition. Power = 0.550**						
**Effect**	**Degr. of Freedom**	**Albumin** **SS**	**Albumin** **MS**	**Albumin** **F**	**Albumin** ***p***	
Intercept	1	27,842.250	27,842.250	458.230	<0.001	
TNM group	5	301.180	60.240	0.991	0.435	
Error	40	2430.420	60.760			
**Tukey HSD test; variable BMI. Approximate Probabilities for Post Hoc Tests, Error: Between MS = 21.973. df = 97.000**						
**TNM group**	**oral cavity**	**p16 neg. oropharynx**	**p16 pos. oropharynx**	**hypopharynx**	**supraglottic larynx**	**glottic larynx**
	24.101	21.722	27.517	25.404	24.941	25.326
oral cavity		0.778	0.556	0.969	0.998	0.979
p16 neg. oropharynx	0.778		0.030	0.110	0.487	0.158
p16 pos. oropharynx	0.556	0.030		0.819	0.807	0.815
hypopharynx	0.969	0.110	0.819		1.000	1.000
supraglottic larynx	0.998	0.487	0.807	1.000		1.000
glottic larynx	0.979	0.158	0.815	1.000	1.000	

**Table 7:** Full Factorial General Linear Model of BMI, PNI status and TNM 8/9 groups for ENE-positivity/negativity. The test headers are highlighted in bold.

Full Factorial General Linear Model: Univariate Results for Each DV, Sigma-Restricted Parameterization, Effective Hypothesis Decomposition						
Effect	ENE+/− Degr. of Freedom	ENE+/− SS	ENE+/− MS	ENE+/− F	ENE+/− *p*	Power (α = 0.05)
Intercept	1	1.343	1.343	5.643	0.020	
BMI	1	0.096	0.096	0.403	0.527	0.096
TNM group	5	2.730	0.546	2.294	0.051	0.718
Error	96	22.848	0.238			
Total	102	25.631				
**Full Factorial General Linear Model: Univariate Results for Each DV, Sigma-restricted parameterization, Effective hypothesis decomposition.**						
**Effect**	**ENE+/−** **Degr. of Freedom**	**ENE+/−** **SS**	**ENE+/−** **MS**	**ENE+/−** **F**	**ENE+/−** ***p***	**Power (α = 0.05)**
Intercept	1	1.046	1.046	4.546	0.039	
PNI	1	0.075	0.075	0.324	0.572	0.086
TNM group	5	1.134	0.227	0.985	0.439	0.313
Error	39	8.975	0.23			
Total	45	10.109				
**Full Factorial General Linear Model: Univariate Results for Each DV, Sigma-restricted parameterization, Effective hypothesis decomposition.**						
**Effect**	**ENE+/−** **Degr. of Freedom**	**ENE+/−** **SS**	**ENE+/−** **MS**	**ENE+/−** **F**	**ENE+/−** ***p***	**Power (α = 0.05)**
Intercept	1	16.937	16.937	74.682	<0.001	
Weight loss	1	0.339	0.339	1.494	0.225	0.227
TNM group	5	3.461	0.692	3.052	0.014	0.848
Error	86	19.504	0.227			
Total	92	23.032				
**Full Factorial General Linear Model: Univariate Results for Each DV. Sigma-restricted parameterization. Effective hypothesis decomposition.**						
**Effect**	**ENE+/−** **Degr. of Freedom**	**ENE+/−** **SS**	**ENE+/−** **MS**	**ENE+/−** **F**	**ENE+/−** ***p***	**Power (α = 0.05)**
Intercept	1	1.533	1.533	6.747	0.013	
Albumin	1	0.191	0.191	0.839	0.365	0.145
TNM group	5	1.218	0.244	1.073	0.390	0.340
Error	39	8.859	0.227			
Total	45	10.109				

**Table 8:** Two-way summary table and Yates’s Chi-square test of tobacco use/alcohol abuse and ENE status. The test headers are highlighted in bold.

2-Way Summary Table of Tobacco Use and ENE Status. Power (α = 0.05) = 0.060			
	ENE-Negative	ENE-Positive	Row Totals
no tobacco use	4	5	9
tobacco use	43	54	97
Totals	47	59	106
Yates Chi-square	Chi-square = 0.118	df = 1	*p* = 0.731
**2-Way Summary Table of alcohol abuse and ENE status. Power (α = 0.05) = 0.230**			
	**ENE-negative**	**ENE-positive**	**Row Totals**
no alcohol abuse	23	19	42
alcohol abuse	25	36	61
Totals	48	55	103
Yates Chi-square	Chi-square = 1.384	df = 1	*p* = 0.239

## Data Availability

The datasets used and analyzed during the current study are available from the corresponding author on reasonable request.

## Abbreviations

The following abbreviations are used in this manuscript:

BMI	Body Mass Index
PNI	Prognostic Nutritional Index
HNSCC	Head and Neck Squamous Cell Carcinoma
ENE	Extranodal extension
